# Phacoemulsification with IOL implantation combined with goniosynechialysis and goniotomy in primary angle-closure glaucoma following failed trabeculectomy: short-term effectiveness and safety outcomes

**DOI:** 10.3389/fmed.2026.1842636

**Published:** 2026-05-18

**Authors:** Lujia Zhou, Huimin Ge, Yangyang Jin, Yan Ma, Li Tang, Weiling Yan, Qing Li, Cheng Lai, Guofan Cao, Xiyan Ding

**Affiliations:** 1 The Affiliated Eye Hospital, Nanjing Medical University, Nanjing, Jiangsu, China; 2 The Fourth School of Clinical Medicine, Nanjing Medical University, Nanjing, China

**Keywords:** failed trabeculectomy, goniosynechialysis, goniotomy, LAN microhook goniotomy knife, phacoemulsification, primary angle-closure glaucoma

## Abstract

**Purpose:**

To evaluate the short-term effectiveness and safety of Phacoemulsification with intraocular lens implantation combined with goniosynechialysis and goniotomy (PEI-GSL-GT) in eyes with prior failed trabeculectomy compared to surgically naïve eyes.

**Materials and methods:**

This retrospective study enrolled 40 eyes (34 patients) diagnosed with moderate to advanced primary angle-closure glaucoma (PACG) who received treatment at the Eye Hospital of Nanjing Medical University from November 2024 to November 2025. After excluding one patient lost to follow-up, 39 eyes (33 patients) with at least 3 months of follow-up were finally included. Among them, 17 cases underwent reoperation due to previous trabeculectomy failure, while 22 cases received the surgical intervention for the first time. All eyes underwent the PEI-GSL-GT procedure. The cohort was divided into a Naïve Group (no prior glaucoma surgery) and a Trabeculectomy failure group (prior failed trabeculectomy). Main outcome measures included intraocular pressure (IOP), best-corrected visual acuity (BCVA), the number of anti-glaucoma medications, and postoperative complications. Surgical success was analyzed using Kaplan-Meier survival curves.

**Results:**

Baseline characteristics were comparable between the Naïve (*n* = 22) and Trabeculectomy failure (*n* = 17) groups (*P* > 0.05). At 3 months, mean IOP decreased significantly in both groups (Naïve: 22.05–14.62 mmHg, *P* = 0.002; Trabeculectomy failure group: 24.53 to 17.00 mmHg, *P* = 0.035), with no significant between-group differences (*P* > 0.05). Medication burden was significantly reduced in both cohorts. Exploratory Kaplan-Meier analysis showed no significant difference in short-term cumulative success (Log-rank *P* = 0.750). Safety profiles were comparable with no vision-threatening complications. BCVA improved in the Naïve Group (*P* < 0.001) and the Trabeculectomy failure group (*P* = 0.177).

**Conclusion:**

PEI-GSL-GT was associated with significant IOP reduction and reduced medication burden during short-term follow-up. No statistically significant differences were observed between the Trabeculectomy failure group and Naïve groups in most outcomes during the 3-month follow-up.

## 1 Introduction

Primary angle-closure glaucoma (PACG) is a leading cause of irreversible blindness (1). While trabeculectomy is the gold standard for medically uncontrolled PACG, its success relies on maintaining a functional filtering bleb; scarring often leads to surgical failure (2). Managing these cases is challenging: medical therapy is often insufficient, repeat trabeculectomy frequently fails due to conjunctival fibrosis, and drainage devices or cyclodestructive procedures carry risks of corneal decompensation or phthisis bulbi (3, 4). Thus, a minimally invasive, safe, and conjunctiva-sparing alternative is urgently needed.

Angle-based surgery offers a potential solution. Phacoemulsification with intraocular lens implantation combined with goniosynechialysis and goniotomy (PEI-GSL-GT) is an emerging treatment for PACG (5, 6). This triple procedure relieves pupillary block via PEI, re-exposes the angle via GSL, and bypasses trabecular resistance via GT, thereby restoring the physiological outflow pathway (7, 8). While its effectiveness is established in surgically naïve PACG (9), its bleb-independent nature preserves the ocular surface for future interventions.

However, evidence for PEI-GSL-GT in glaucoma with failed filtering surgery remains limited. Concerns persist that distal outflow pathways (e.g., Schlemm’s canal) in these eyes may be compromised by chronic high IOP or prior surgery, potentially limiting the effectiveness of angle-based procedures (8, 9). To address this gap, this study evaluates the short-term effectiveness and safety of PEI-GSL-GT in eyes with failed trabeculectomy compared with surgically naïve eyes.

## 2 Materials and methods

### 2.1 Design, setting, and participants

This retrospective, comparative case series was conducted at the Eye Hospital of Nanjing Medical University. The study protocol adhered to the tenets of the Declaration of Helsinki and was approved by the Institutional Review Board (IRB) (Ethics Number: NO.2023021). Written informed consent was obtained from all participants prior to surgery.

**FIGURE 1:**
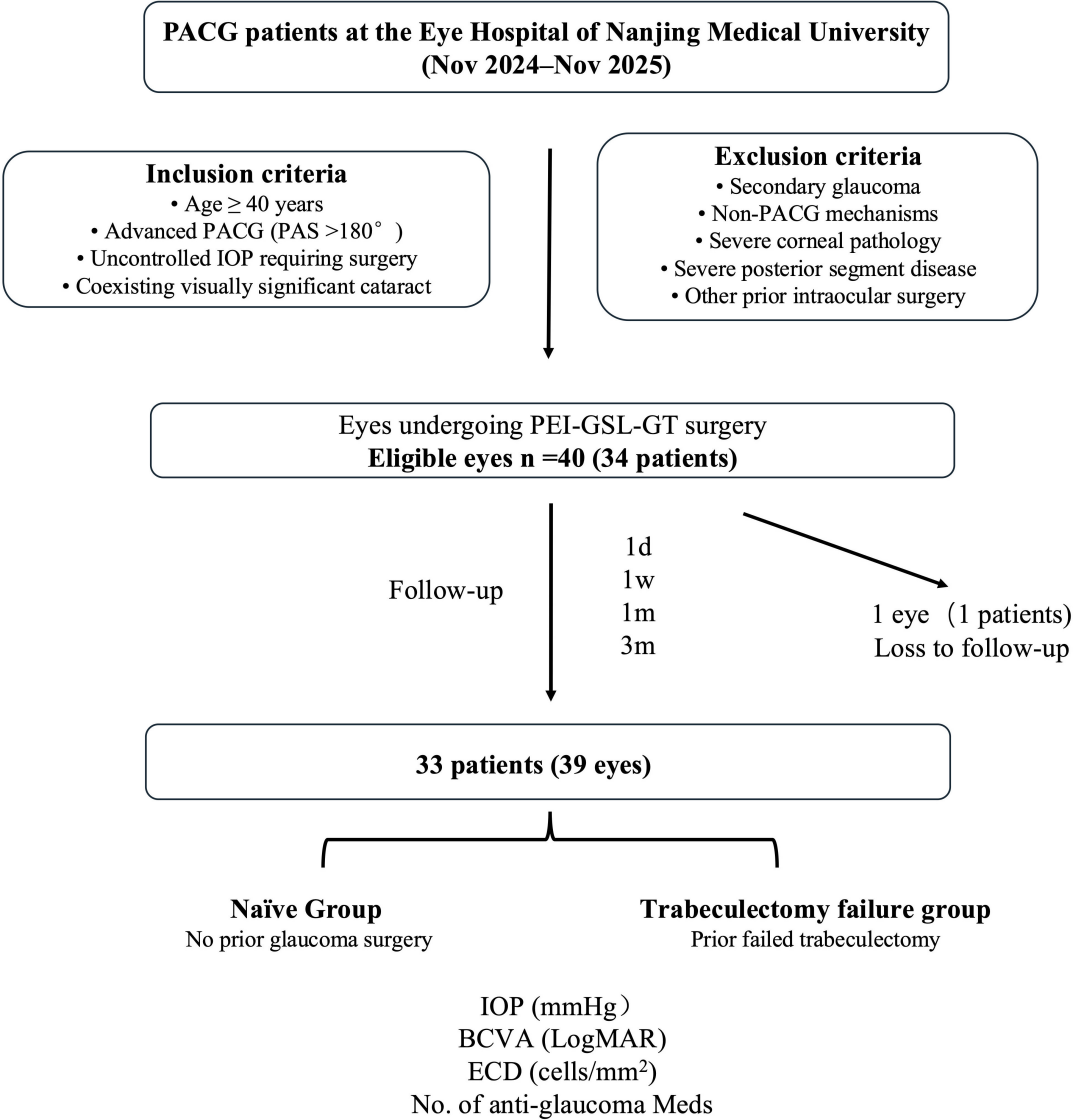
Flowchart of patient selection. A total of 34 patients (40 eyes) with moderate-to-advanced primary angle-closure glaucoma and coexisting cataract who underwent PEI-GSL-GT were screened. After exclusion of 1 patient (1 eye) with follow-up of less than 3 months, 33 patients (39 eyes) were included in the final analysis, including 22 eyes in the Naïve Group and 17 eyes in the Trabeculectomy failure group.

We reviewed the medical records of patients with advanced PACG and coexisting cataracts who underwent PEI-GSL-GT between November 2024 and November 2025 (Figure 1). The inclusion criteria were as follows: (1) age ≥ 40 years; (2) medically uncontrolled IOP requiring surgical intervention despite maximal tolerated medical therapy; (3) diagnosis of PACG, defined as gonioscopic evidence of peripheral anterior synechiae (PAS) > 180 degrees (involving at least the nasal or inferior quadrant), accompanied by glaucomatous optic neuropathy (GON) and characteristic visual field defects (e.g., nasal step, arcuate scotoma, or paracentral scotoma); and (4) presence of visually significant cataract (10). Patients were stratified into two groups based on surgical history: the Naïve Group (eyes without prior glaucoma surgery) and the Trabeculectomy failure group (eyes that had undergone at least one prior trabeculectomy and subsequently developed a flattened, encapsulated, or nonfunctioning filtering bleb, with IOP remaining above target despite maximally tolerated medical therapy and requiring reoperation) (11).

The exclusion criteria included: (1) secondary glaucoma (e.g., uveitic, neovascular, traumatic, or lens-induced); (2) plateau iris configuration or other non-pupillary block/non-PAS mechanisms; (3) severe corneal pathology (e.g., leukoma or edema) precluding intraoperative visualization of the angle; (4) severe posterior segment pathology affecting visual potential (e.g., proliferative diabetic retinopathy, central retinal vein occlusion); and (5) history of other intraocular surgeries (e.g., vitrectomy, tube shunt implantation) other than trabeculectomy in the Trabeculectomy failure group .

### 2.2 Preoperative assessment

All patients underwent a comprehensive preoperative ophthalmic examination. The assessment included: best-corrected visual acuity (BCVA), measured using a Snellen chart and converted to logarithm of the minimum angle of resolution (LogMAR) for statistical analysis; IOP, measured using a Non-contact Tonometer (NCT), with the IOP value recorded as the average of three consecutive readings taken by the same experienced ophthalmologist; slit-lamp biomicroscopy and fundus examination to evaluate the health of the anterior and posterior segments of the eye; gonioscopy using a Goldmann three-mirror lens to record the extent of PAS; standard automated perimetry (Humphrey Field Analyzer, Carl Zeiss Meditec) to assess the MD of the visual field; and corneal endothelial cell density (ECD) measured by specular microscopy.

### 2.3 Surgical technique

All surgeries were performed by the same experienced glaucoma specialist under local anesthesia.

PEI: A clear corneal incision was created in the superonasal quadrant. If posterior synechiae were present, an iris repositor was used to separate the iris from the anterior lens capsule. Standard phacoemulsification was performed to remove the cataractous lens, followed by the implantation of a foldable IOL into the capsular bag. GSL and GT: Following IOL implantation, an ophthalmic viscosurgical device (OVD) was injected to deepen the anterior chamber and widen the angle. The surgical microscope was tilted approximately 35° nasally, and a surgical gonioscope was applied. GSL: An iris repositor was used to mechanically strip the PAS downward, exposing the trabecular meshwork. GT: Under gonioscope visualization, goniotomy was performed using LAN microhook goniotomy knife (LAN microhook). The incision of the trabecular meshwork spanned approximately 120°, covering the inferior, inferonasal, and inferotemporal quadrants.

Finally, the OVD was thoroughly aspirated from the anterior chamber. The incisions were hydrated and sutured if necessary to ensure watertight closure.

### 2.4 Postoperative management

Postoperatively, all patients received a standardized medication regimen. Topical antibiotic therapy consisting of 0.5% levofloxacin (three times daily) combined with tobramycin-dexamethasone eye drops (four times daily) was administered for 1 week. Subsequently, the corticosteroid dosage was gradually tapered over a 4-week period. To prevent the recurrence of PAS and maintain the patency of the anterior chamber angle, compound tropicamide eye drops were prescribed twice daily for 1 month. Additionally, sodium hyaluronate eye drops were applied to lubricate the ocular surface, and oral mecobalamin and citicoline sodium tablets were administered to provide neurotrophic support to the optic nerve.

Follow-up visits were scheduled at 1 day, 1 week, 1 month, and 3 months postoperatively. Examinations at each visit included BCVA, slit-lamp biomicroscopy, and NCT. Management of IOP Spikes: Transient IOP elevation ( > 21 mmHg) in the early postoperative period was managed by resuming anti-glaucoma medications. In cases of significant acute elevation (e.g., on postoperative day 1), anterior chamber paracentesis was performed at the slit lamp to rapidly lower IOP and protect the optic nerve.

### 2.5 Outcome measures and definitions

The primary outcome measure was the surgical success rate, with the cumulative success rate calculated using Kaplan-Meier survival analysis. Secondary outcome measures included the mean postoperative intraocular pressure, the number of anti-glaucoma medications, visual acuity (BCVA), and the incidence of intraoperative and postoperative complications.

Definition of surgical success: Surgical success was divided into two grades: Qualified Success: defined as a postoperative intraocular pressure ≤ 21 mmHg with a reduction of ≥ 20% from baseline, regardless of the use of anti-glaucoma medications. Complete Success: defined as meeting the above criteria for qualified success without the use of any anti-glaucoma medications. Criteria for eyes with low baseline intraocular pressure: For eyes with preoperatively controlled intraocular pressure (≤21 mmHg) under maximum medical treatment, success was defined as maintaining an intraocular pressure ≤ 21 mmHg with a reduction in the number of anti-glaucoma medications compared to baseline.

Definition of failure: An eye was classified as a surgical failure if it met any of the following criteria: Uncontrolled intraocular pressure: After 1 month postoperatively, two consecutive follow-ups showed an intraocular pressure > 21 mmHg or a reduction in intraocular pressure < 20%. Reoperation: any surgical treatment performed to control intraocular pressure. Visual loss: loss of light perception. Note on transient intraocular pressure elevation: Transient intraocular pressure elevation occurring within the first month postoperatively (e.g., due to residual viscoelastic agent), if resolved with medication or anterior chamber paracentesis, was not classified as surgical failure. The time of failure was recorded as the date of the first visit that met the failure criteria.

### 2.6 Statistical analysis

Statistical analyses were performed using SPSS version 27.0 (IBM Corp., Armonk, NY, United States). Because treatment group assignment was defined at the eye level and both eyes from the same patient could be included, generalized estimating equations (GEE) were used to account for inter-eye correlation, with patient specified as the clustering variable and an exchangeable working correlation structure applied. Continuous outcomes were analyzed using a normal distribution with identity link, binary outcomes using a binomial distribution with logit link, and count outcomes using Poisson or negative binomial distributions, as appropriate. Longitudinal analyses of IOP, BCVA, and medication use included group, time, and group-by-time interaction terms. Data are presented as mean ± standard deviation (SD) or median (interquartile range, IQR), as appropriate. Postoperative complications were summarized descriptively. Kaplan–Meier survival analysis was used as an exploratory description of short-term surgical success, and survival curves were compared using the log-rank test. Comparisons of sparse categorical complications were considered exploratory and assessed using Fisher’s exact test where applicable. A two-sided *P* < 0.05 was considered statistically significant.

## 3 Results

### 3.1 Baseline characteristics

A total of 40 eyes from 34 patients with PACG and coexisting cataract requiring surgical IOP control were initially enrolled. One patient was lost to follow-up, and 33 patients (39 eyes) with at least 3 months of follow-up were available for the final analysis. The cohort comprised 22 eyes in the Naïve Group and 17 eyes in the Trabeculectomy failure group, defined as eyes with prior failed trabeculectomy. Baseline demographic, ocular biometric, and clinical characteristics are summarized in Table 1. The two groups were generally comparable in age, sex distribution, baseline IOP, medication burden, visual field mean deviation, and anterior segment biometric parameters. However, baseline BCVA was worse and ECD was lower in the Trabeculectomy failure group. Despite this difference, preoperative ECD remained within a clinically acceptable range in both groups. Overall, the two groups were broadly comparable at baseline, except for worse visual acuity and lower ECD in the Trabeculectomy failure group.

**TABLE 1:** Preoperative demographic, ocular biometric, and clinical characteristics.

Variable	Naïve group (*n* = 22)	Trabeculectomy failure group (*n* = 17)	*P*-value
Age (years)	69.64 ± 6.336	68.29 ± 8.447	0.195
Sex (Female, n%)	12 (54.5%)	12(70.6%)	0.529
Baseline IOP (mmHg)	22.05 ± 11.35	24.53 ± 13.187	0.598
BCVA (LogMAR)	0.699 (0.30, 1.00)	0.92 (0.39, 1.85)	0.042
ECD (cells/mm^2^)	2674 (2478, 2855)	2527 (1122, 2638)	0.018
No. of anti-glaucoma Meds	2 (1, 3)	2(1, 2)	0.353
Visual field MD (dB)	−18.51 (−28.29, −9.34)	−19.11 (−28.22, −9.06)	0.245
Axial Length (mm)	22.79 (22.29, 23.07)	22.29 (21.66, 23.04)	0.155
Anterior chamber depth (mm)	2.39 (2.10, 2.78)	2.14 (1.77, 2.37)	0.156
CCT (μm)	547 (519, 567)	553 (539.5, 594)	0.07
Lens thickness (mm)	4.48 ± 1.13	4.18 ± 1.32	0.468
LOCS III nuclear color (NC)	3 (2, 3)	3 (2,3)	0.612
PAS extent (quadrants)	4 (3, 4)	4 (2, 4)	0.384

Data are presented as mean ± SD, median (IQR), or n (%), as appropriate. *P*-values were derived from generalized estimating equation (GEE) models with patient as the clustering variable to account for inter-eye correlation. BCVA, Best-corrected visual acuity; ECD, Endothelial Cell Density; AL, axial length; CCT, central corneal thickness; PAS, Peripheral Anterior Synechiae.

In the Trabeculectomy failure group, most eyes had undergone one prior trabeculectomy, whereas a few eyes had undergone two prior procedures. The interval between prior trabeculectomy and the current surgery varied widely, with a median of 24 months (range, 2–270 months). Nine patients underwent trabeculectomy in our hospital with intraoperative application of mitomycin C, while the surgical details of the remaining patients were unknown. Nevertheless, all filtering blebs appeared flat and non-functional before reoperation.

### 3.2 Intraocular pressure outcomes

IOP decreased in both groups during the 3-month follow-up after PEI-GSL-GT. In the Naïve Group, mean IOP decreased from 22.05 ± 11.35 mmHg preoperatively to 14.62 ± 2.99 mmHg at 3 months (*P* = 0.002). Similarly, in the Trabeculectomy failure group, mean IOP decreased from 24.53 ± 13.19 mmHg to 17.00 ± 6.09 mmHg at 3 months (*P* = 0.035).

**FIGURE 2:**
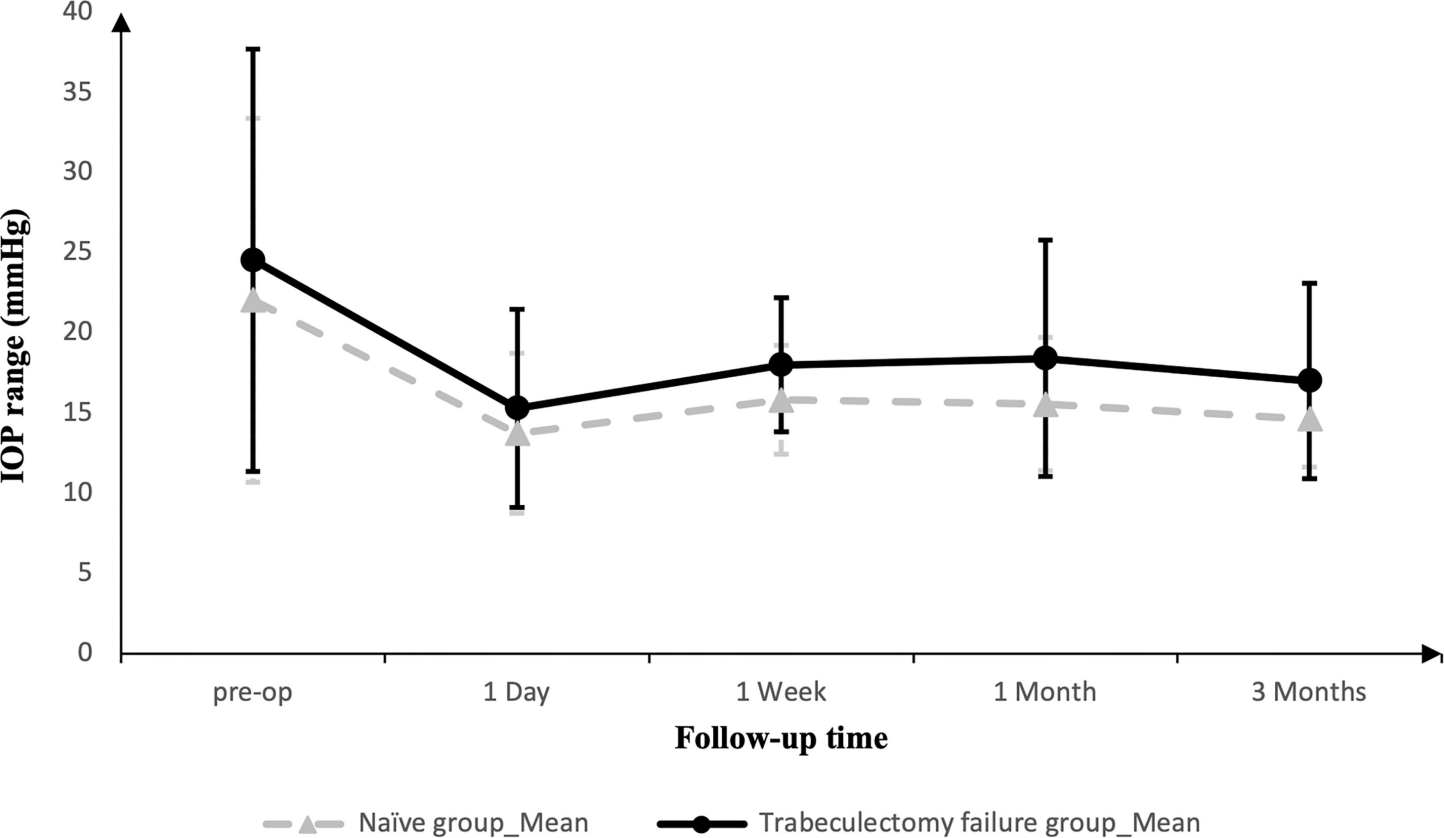
Longitudinal profile of mean intraocular pressure (IOP). Line graph comparing the mean IOP at preoperative baseline and postoperative follow-up visits (1 day, 1 week, 1 and 3 months) between the Naïve Group and the Trabeculectomy failure group. Error bars represent standard deviations.

The longitudinal IOP profile is shown in Figure 2. The Naïve Group showed a relatively steady postoperative decline in IOP, whereas the Trabeculectomy failure group showed a mild transient increase at 1 month, followed by stabilization at 3 months. Between-group comparisons showed no statistically significant difference in IOP at baseline, postoperative day 1, 1 month, or 3 months; however, IOP was lower in the Naïve Group at 1 week postoperatively. Overall, lower postoperative IOP values were observed in both groups during short-term follow-up.

**FIGURE 3:**
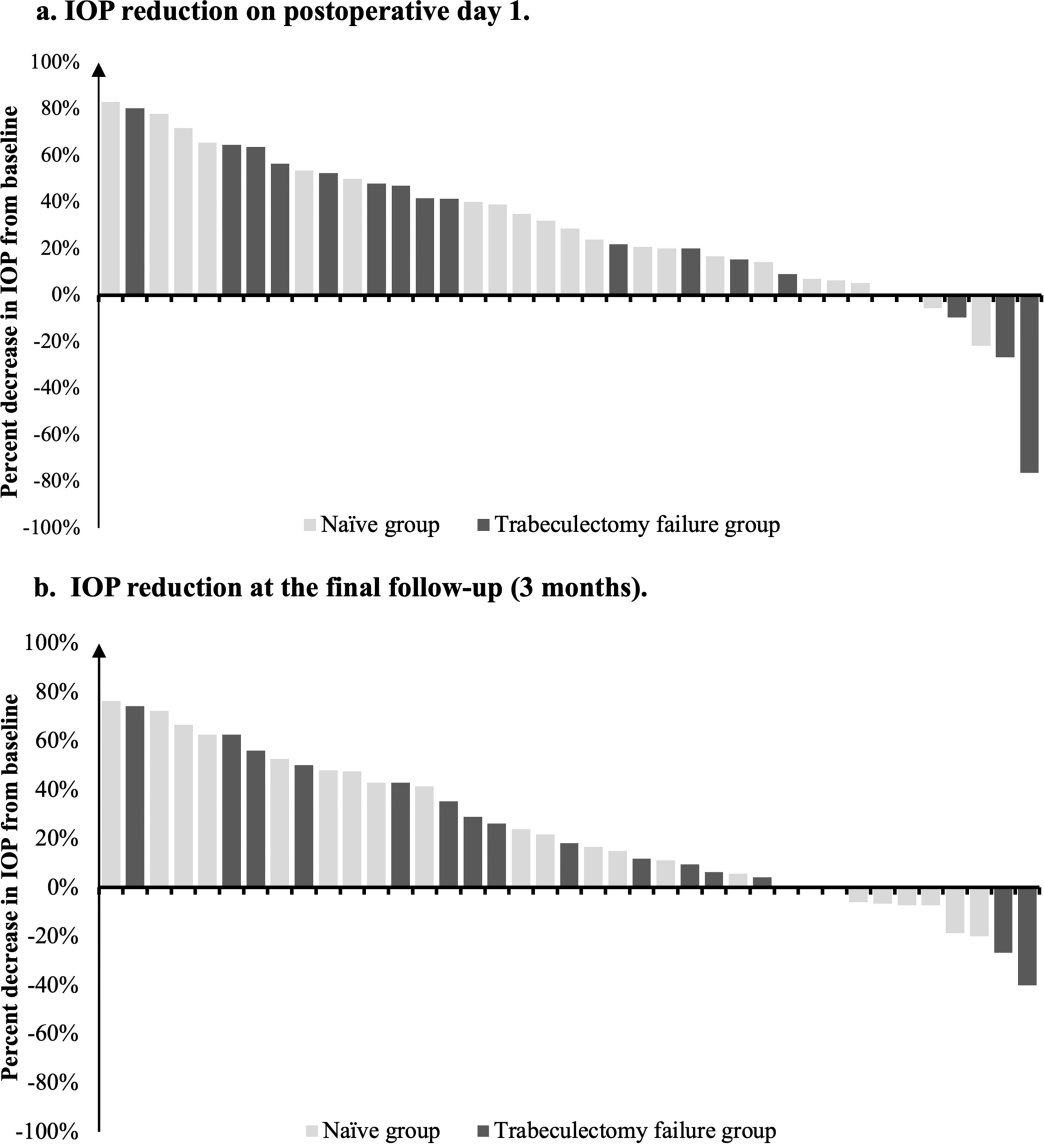
Distribution of individual percentage IOP reduction. Waterfall plots showing the percentage reduction in intraocular pressure (IOP) from baseline for each individual eye in the Naïve Group (gray bars) and Trabeculectomy failure group (black bars). **(a)** IOP reduction on postoperative day 1. **(b)** IOP reduction at the final follow-up (3 months). Bars above the 0% line indicate a reduction in IOP, while bars below the line indicate an increase in IOP compared to baseline.

Individual percentage IOP reduction is illustrated in the waterfall plots in Figure 3. On postoperative day 1 (Figure 3a), most eyes in both groups showed an immediate reduction in IOP. The Naïve Group demonstrated a relatively consistent early response, whereas the Trabeculectomy failure group showed greater heterogeneity, with a few eyes showing transient IOP elevation. By 3 months (Figure 3b), the distribution of IOP reduction appeared more homogeneous, and most eyes in both groups maintained lower IOP than at baseline. These plots indicate a general trend toward maintained IOP reduction during short-term follow-up, despite inter-eye variability.

### 3.3 Reduction in medication burden

**FIGURE 4:**
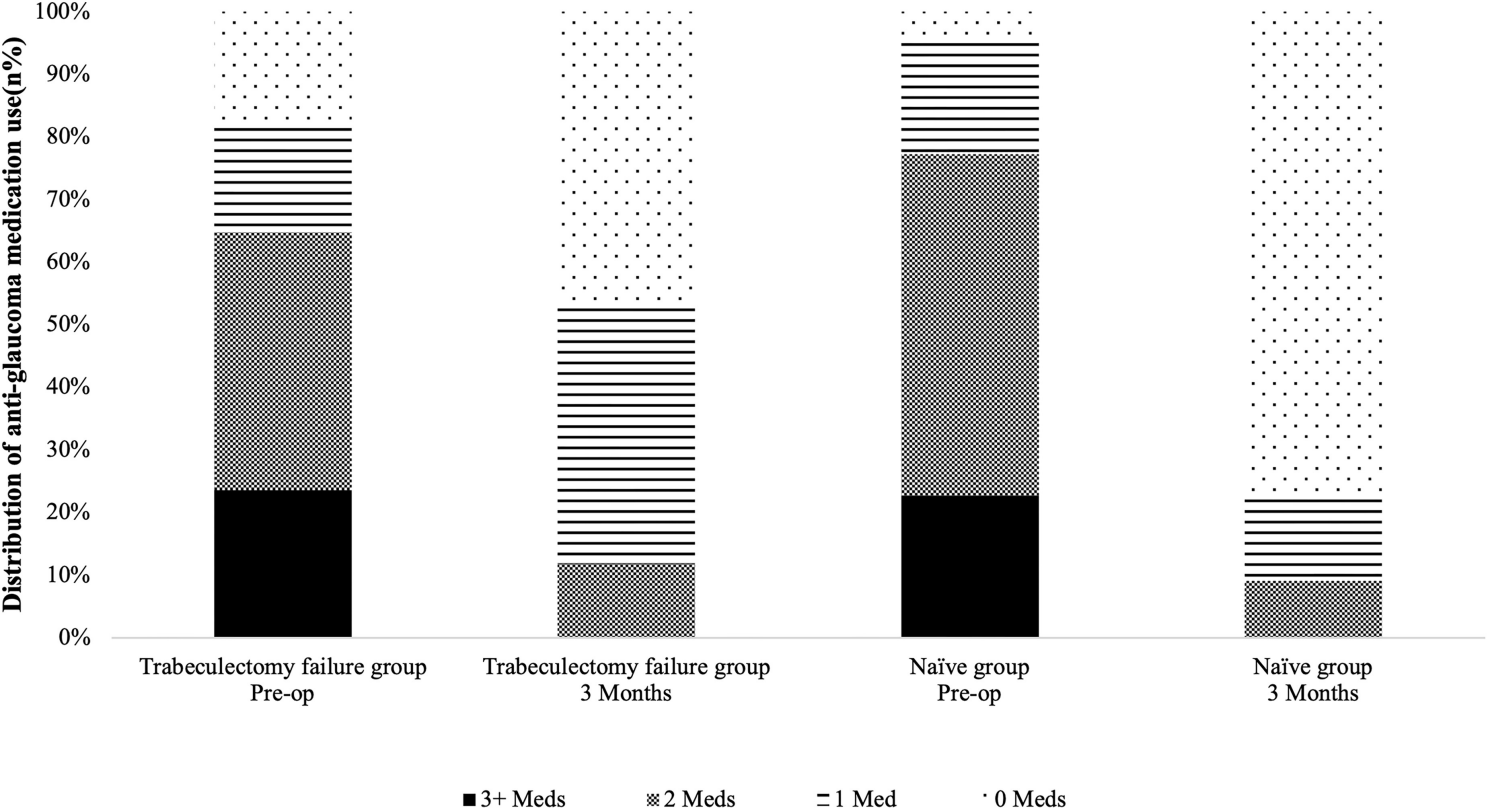
Changes in the number of glaucoma medications. Stacked bar chart illustrating the proportion of eyes requiring 0, 1, 2, or ≥ 3 anti-glaucoma medications at baseline (Pre-op) and at the 3-month postoperative visit.

PEI-GSL-GT was associated with a reduction in medication burden in both groups (Table 2). Baseline medication use was comparable between the Naïve and Trabeculectomy failure group (*P* = 0.353). At 3 months, the median number of anti-glaucoma medications had decreased to 0 in the Naïve Group and 1 in the Trabeculectomy failure group. Postoperative medication requirements at 3 months did not differ significantly between groups (*P* = 0.525). As shown in Figure 4, the proportion of medication-free eyes increased markedly in both groups, accompanied by a reduction in the proportion of eyes requiring multiple medications ( ≥ 2).

**TABLE 2:** Comparison of IOP, medications, and BCVA over time.

Time point	Naïve group (*n* = 22)	Trabeculectomy failure group (*n* = 17)	*P*-value*
Preoperative			
IOP (mmHg)	22.05 ± 11.35	24.53 ± 13.19	0.532
Meds (n)	2 (1, 3)	2 (1, 2)	0.353
BCVA (LogMAR)	0.699 (0.30, 1.00)	0.92 (0.39, 1.85)	0.042
1 Day postop			
IOP (mmHg)	13.73 ± 4.99	15.29 ± 6.17	0.38
Meds (n)	0 (0, 1)	0 (0, 0)	0.325
1 week postop			
IOP (mmHg)	15.82 ± 3.40	18.00 ± 4.17	0.017
Meds (n)	0 (0, 1)	0 (0, 1)	0.228
1 month postop			
IOP (mmHg)	15.55 ± 4.17	18.41 ± 7.38	0.14
Meds (n)	0 (0, 1)	0.5 (0, 1.25)	0.322
3 months postop			
IOP (mmHg)	14.62 ± 2.99	17.00 ± 6.09	0.099
Meds (n)	0 (0, 0.25)	1 (0, 1)	0.151
BCVA (LogMAR)	0.22 (0.09, 0.51)	0.50 (0.09, 1.00)	0.067
Absolute reduction of IOP (mmHg)	7.64 ± 12.18	7.53 ± 12.95	0.978
Percentage reduction of IOP (%)	18.63 ± 44.61	21.12 ± 30.62	0.832
*P*-value†	0.002	0.035	–
*P*-value^†⁣†^	< 0.001	0.023	–

Data are presented as mean ± SD or median (IQR), as appropriate. *P-*value represents between-group comparisons at each time point using generalized estimating equations (GEE) to account for inter-eye correlation. *P*-value† and *P*-value†† represent within-group comparisons between baseline and 3 months for IOP and BCVA, respectively, based on GEE models. *P* Value† indicates 3 months IOP compared with preoperative baseline IOP; Value†† Indicates 3 months BCVA compared with preoperative baseline. (Longitudinal comparisons were performed using Paired *t*-test for IOP and Wilcoxon signed-rank test for IOP/BCVA). *Represent statistically significant differences in characteristics between the Naïve Group and the Trabeculectomy failure group.

### 3.4 Surgical success rate

At 3 months, qualified success was achieved in (19/22) eyes [(86.4%)] in the Naïve Group and (14/17) eyes [(82.4%)] in the Trabeculectomy failure group. Complete success was achieved in (20/22) eyes [(90.9%)] and (13/17) eyes [(76.5%)], respectively. Surgical failure occurred in (2/22) eyes [(9.10%)] in the Naïve Group and (4/17) eyes [(23.5%)] in the Trabeculectomy failure group.

**FIGURE 5:**
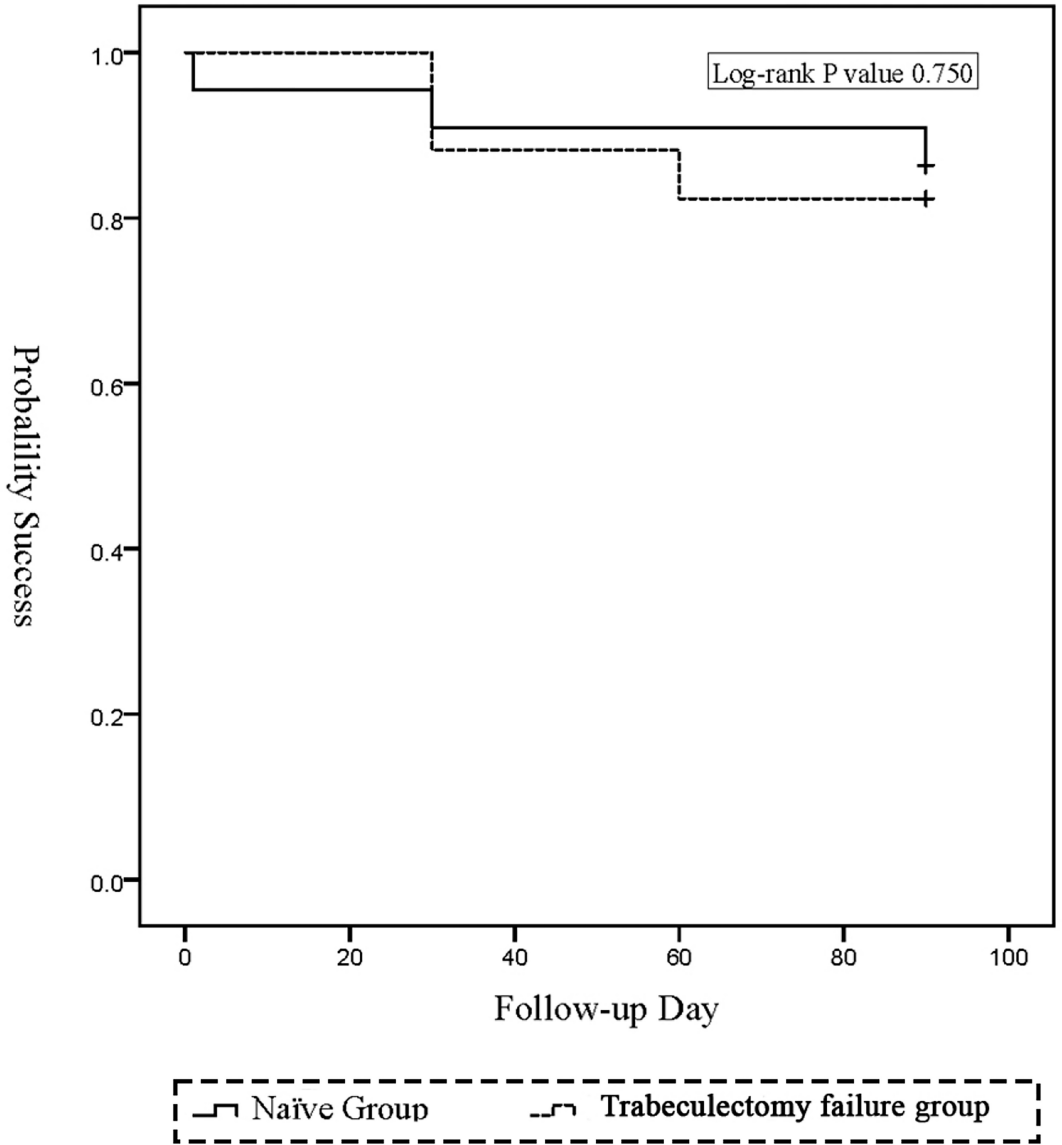
Kaplan-Meier survival analysis of surgical success. The graph compares the cumulative probability of qualified success between the Naïve Group (dashed line) and Trabeculectomy failure group (solid line) over the 3-month follow-up period. Qualified success was defined as an IOP ≤ 21 mmHg and ≥ 20% reduction from baseline, with or without medications. Failure was defined as not meeting these criteria on two consecutive visits or requiring further glaucoma surgery.

Kaplan–Meier analysis was additionally performed as an exploratory description of short-term success over time (Figure 5). The log-rank test showed no significant difference between the two groups (*P* = 0.750). These exploratory findings suggest that the short-term success profile of PEI-GSL-GT in eyes with prior failed filtering surgery may be broadly similar to that in surgically naïve eyes.

### 3.5 Visual acuity and safety profile

Visual acuity outcomes are detailed in Table 2. Following cataract extraction, the Naïve Group experienced a statistically significant improvement in median BCVA from 0.699 to 0.22 (*P* < 0.001). In the Trabeculectomy failure group, median BCVA improved from 0.92 to 0.50 (*P* = 0.023), indicating that the procedure did not compromise visual function in these high-risk eyes.

Postoperative complications are summarized in Table 3. Overall, the safety profile was favorable in both groups, and no sight-threatening complications were observed. Because postoperative adverse events were infrequent, comparisons were considered exploratory. IOP spikes (>21 mmHg) occurred in 2 eyes (11.8%) in the Trabeculectomy failure group and in none of the eyes in the Naïve Group; both cases resolved after aqueous release on postoperative day 1. Minor complications, including hyphema and corneal edema, were uncommon and resolved with conservative management.

**TABLE 3:** Comparison of complications between groups.

Complications, n(%)	Naïve (*n* = 22)	Trabeculectomy failure group (*n* = 17)	*P*-value
Hyphema	3 (13.6%)	1 (5.9%)	0.618
IOP spikes ( > 21 mmHg)	0 (0%)	2 (11.8%)	0.184
Corneal oedema	1 (4.5%)	2 (11.8%)	0.570
Serious complications	0 (0%)	0 (0%)	NA

Data are presented as n (%). Owing to the low event frequency, comparisons were considered exploratory and performed using Fisher’s exact test where applicable.

## 4 Discussion

In this study, we confirmed that PEI-GSL-GT holds certain potential in the treatment of PACG with coexisting cataract, which can reduce intraocular pressure and alleviate medication burden in the short term (12, 13). A pivotal finding of our investigation is that short-term therapeutic effectiveness in eyes with failed trabeculectomy was comparable to that in surgically naïve eyes (14). These findings suggest that this angle-based triple procedure may serve as a feasible rescue option in selected eyes with failed filtering surgery.

PEI-GSL-GT simultaneously addresses three distinct levels of aqueous outflow obstruction: phacoemulsification relieves pupillary block and deepens the anterior chamber (15–17); GSL mechanically releases PAS to expose the angle (18); and GT bypasses the resistance at the trabecular meshwork (7, 8). The synergistic value of this triple combination is evident when compared to single or dual procedures reported in previous literature (9, 19). While PEI-GSL alone effectively opens the angle, it fails to address the intrinsic trabecular resistance often elevated in chronic glaucoma. Conversely, standalone goniotomy may be technically unfeasible if extensive PAS obscures the trabecular meshwork (8, 20). By integrating these three steps, our protocol maximizes the recruitment of the outflow pathway, which is particularly crucial for complex cases with mixed mechanisms of obstruction (9, 21).

Regarding the baseline characteristics, it is noteworthy that the mean preoperative IOP in our cohort appeared relatively controlled (approximately 20–22 mmHg). This value, however, must be interpreted in the context of maximum medical therapy. All patients were maintained on a full regimen of anti-glaucoma medications up to the day of surgery to prevent optic nerve damage. Therefore, the severity of the disease in our cohort is reflected not merely by the raw preoperative IOP values, but by the heavy medication burden (median of 2–3 classes) required to maintain these levels.

Previous studies indicate that traditional trabeculectomy may achieve lower absolute IOP levels (often in the low teens) compared to angle-based procedures (22). However, this superior effectiveness is associated with a higher risk of vision-threatening complications (23). In our study, PEI-GSL-GT achieved a mean IOP of approximately 16 mmHg in the Trabeculectomy failure group without serious complications. The most common complication observed was mild hyphema, typically occurring on the first postoperative day. This is a common and anticipated sequela of goniotomy, resulting from blood reflux through the incised Schlemm’s canal. Importantly, all cases of hyphema were self-limiting, resolving spontaneously within a few days without compromising visual outcomes (24). For the elderly cohort in our study, this represents a favorable trade-off: prioritizing a superior safety profile and rapid visual rehabilitation over the pursuit of extremely low IOP (25, 26).

Regarding the postoperative course, transient IOP fluctuations were observed in the early phase, particularly around the 1-month follow-up. Such IOP peaks were self-limiting and responded well to medical treatment in our cohort, although longer follow-up is still required to determine their long-term significance. Potential mechanisms for these early fluctuations include a steroid-response IOP elevation due to postoperative anti-inflammatory regimen or transient trabecular edema (trabeculitis) induced by surgical manipulation (27). In analyzing the surgical failures (two cases in the Naïve Group), we hypothesize mechanisms such as recurrent PAS or unrecognized distal outflow resistance (e.g., sclerosis of collector channels) (28). Although our Trabeculectomy failure group showed a high success rate, it is theoretically possible that eyes with a longer duration of failed filtration or more severe glaucomatous damage might exhibit irreversible distal vessel closure. This distinction is critical: PEI-GSL-GT can resolve proximal resistance, but its effectiveness is limited if the downstream drainage system is completely fibrotic (29). Another important safety concern relates to corneal endothelial cell loss. Although corneal endothelial cell density was not remeasured during the short 3-month follow-up, we paid particular attention to endothelial protection by using ophthalmic viscosurgical devices intraoperatively. Only a few patients developed corneal edema on postoperative day 1, and all corneas remained clear throughout follow-up. No severe complications of corneal endothelial decompensation occurred in all cases. To some extent, these findings suggest a favorable short-term corneal safety profile of this quadruple procedure.

A critical controversy for t eyes with failed trabeculectomy concerns the functional integrity of the distal outflow pathway. It has been hypothesized that chronic high IOP and long-term topical medication use may lead to Schlemm’s canal collapse or collector channel atrophy, potentially rendering angle-based procedures ineffective (9, 30). In the present study, no statistically significant difference in short-term IOP reduction was observed between the trabeculectomy failure and Naïve groups during the 3-month follow-up, except at the 1-week visit. These findings may indicate that, in at least some eyes with prior failed filtering surgery, the distal outflow pathway remains functionally recruitable. However, this interpretation is indirect and based on clinical outcomes rather than direct visualization or physiological assessment. In eyes with less favorable outcomes, distal outflow impairment remains a possible explanation. Further studies incorporating longer follow-up and direct evaluation of outflow pathway status are needed to better identify eyes that may benefit from angle-based rescue procedures (31).

Our study has several limitations:

1. *Study design:* The retrospective nature introduces potential selection bias.
2. *Lack of a parallel control group:* Ideally, a prospective comparison with a parallel control group undergoing standard rescue therapies (e.g., Ahmed valve implantation or repeat trabeculectomy) would provide stronger evidence of relative effectiveness. Current evidence does not confirm the advantage of PEI-GSL-GT relative to existing standard therapies. Further follow-up and investigations will be conducted in future studies (32).
3. *Indirect assessment:* We did not utilize imaging techniques (e.g., aqueous angiography or anterior segment OCT) to functionally evaluate the distal outflow tract. Future incorporation of these modalities could help identify biomarkers for surgical success (33, 34).
4. *Outcome metrics*: Primary endpoints were limited to IOP and medication reduction. Due to the short follow-up (3 months), we could not meaningfully analyze changes in visual field (MD) or RNFL thickness, which are the ultimate indicators of neuroprotection.
5. *Long-term durability:* As with any goniotomy-based procedure, there is a risk that the incised trabecular cleft may undergo fibrosis and closure over time. Long-term follow-up is essential to verify whether the IOP-lowering effect is sustained beyond the immediate postoperative period.
6. *Corneal endothelial assessment:* We did not reassess corneal endothelial cell density during the 3-month postoperative follow-up; therefore, the longer-term endothelial safety of this procedure remains to be clarified.

1. *IOP measurement*: The non-contact tonometer enables rapid, noninvasive measurement and facilitates repeated follow-up examinations. To ensure the comparability of IOP data, all patients in this study underwent IOP measurement using the same non-contact tonometer. Nevertheless, Goldmann applanation tonometry remains the clinical gold standard, which represents a major limitation of the present study.

Despite these limitations, this short-term study shows that PEI-GSL-GT can reduce IOP and medication burden in cataractous PACG eyes, even after failed trabecular surgery. In the present cohort, no statistically significant difference in most short-term outcomes was observed between the Naïve and Trabeculectomy failure group during the 3-month follow-up. Larger studies with longer follow-up are needed to further define the durability, safety, and appropriate indications of this procedure.

## Data Availability

The raw data supporting the conclusions of this article will be made available by the authors, without undue reservation.
